# Identification of 9 key genes and small molecule drugs in clear cell renal cell carcinoma

**DOI:** 10.18632/aging.102161

**Published:** 2019-08-18

**Authors:** Yongwen Luo, Dexin Shen, Liang Chen, Gang Wang, Xuefeng Liu, Kaiyu Qian, Yu Xiao, Xinghuan Wang, Lingao Ju

**Affiliations:** 1 Department of Urology, Zhongnan Hospital of Wuhan University, Wuhan, China; 2 Department of Biological Repositories, Zhongnan Hospital of Wuhan University, Wuhan, China; 3 Human Genetics Resource Preservation Center of Hubei Province, Wuhan, China; 4 Laboratory of Precision Medicine, Zhongnan Hospital of Wuhan University, Wuhan, China; 5 Department of Pathology, Lombardi Comprehensive Cancer Center, Georgetown University Medical School, Washington, DC 20007, USA; 6 Medical Research Institute, Wuhan University, Wuhan, China

**Keywords:** clear cell renal cell carcinoma (ccRCC), biomarker, Weighted Gene Co-expression Network Analysis (WGCNA), Gene Set Enrichment Analysis (GSEA)

## Abstract

Clear cell renal cell carcinoma (ccRCC) is a heterogeneous tumor that the underlying molecular mechanisms are largely unclear. This study aimed to elucidate the key candidate genes and pathways in ccRCC by integrated bioinformatics analysis. 1387 differentially expressed genes were identified based on three expression profile datasets, including 673 upregulated genes and 714 downregulated genes. Then we used weighted correlation network analysis to identify 6 modules associated with pathological stage and grade, blue module was the most relevant module. GO and KEGG pathway analyses showed that genes in blue module were enriched in cell cycle and metabolic related pathways. Further, 25 hub genes in blue module were identified as hub genes. Based on GEPIA database, 9 genes were associated with progression and prognosis of ccRCC patients, including PTTG1, RRM2, TOP2A, UHRF1, CEP55, BIRC5, UBE2C, FOXM1 and CDC20. Then multivariate Cox regression showed that the risk score base on 9 key genes signature was a clinically independent prognostic factor for ccRCC patients. Moreover, we screened out several new small molecule drugs that have the potential to treat ccRCC. Few of them were identified as biomarkers in ccRCC. In conclusion, our research identified 9 potential prognostic genes and several candidate small molecule drugs for ccRCC treatment.

## INTRODUCTION

Renal cell carcinoma is common urinary malignancy, which accounts for about 3% of all malignant tumors. In the urinary system, the incidence rate is second to the bladder cancer. According to global cancer statistics 2018, around 403,262 (2.2%) new cases of kidney cancer are diagnosed, and approximately 175,098 (1.8%) died of the disease [1]. Clear cell renal cell carcinoma (ccRCC) is the most common subtype of renal cell carcinoma. It accounts for approximately 80%-90% of renal cell carcinoma [2].

Currently, about 30% of patients are diagnosed with disease that is already in the metastatic stage [3]. For patients with advanced ccRCC or cancer recurrence, a number of molecule-targeted drugs have been used as clinical first-line therapy, including sorafenib, sunitinib, aldesleukin, axitinib and bevacizumab. Compared with chemoradiotherapy, the survival time has been greatly improved. However, because of the side effects of molecule-targeted drugs and individual differences of patient sensitivity to drugs, median disease-free and overall survival times of patients remain short [4]. Therefore, it is of great significance to further explore more effective prognostic biomarkers and therapeutic targets.

With the popularization and gradual development of gene chips and high-throughput sequencing, it is possible to identify the key genes associated with tumor progression and prognosis based on big data integration and bioinformatics. Weighted gene co-expression network analysis (WGCNA) is a systematic biological method that could identify highly synergistically altered gene sets, and based on the intrinsic properties of gene sets and correlation between gene sets and phenotypes, candidate biomarker genes or therapeutic targets can be screened out. The aim of this study was to identify and validate key genes that were significantly associated with oncogenesis and progression in ccRCC tumors by weighted correlation network analysis, and further screen correlated small molecule target drugs.

## RESULTS

### Differentially expressed genes (DEGs) screening of ccRCC

**Figure 1:**
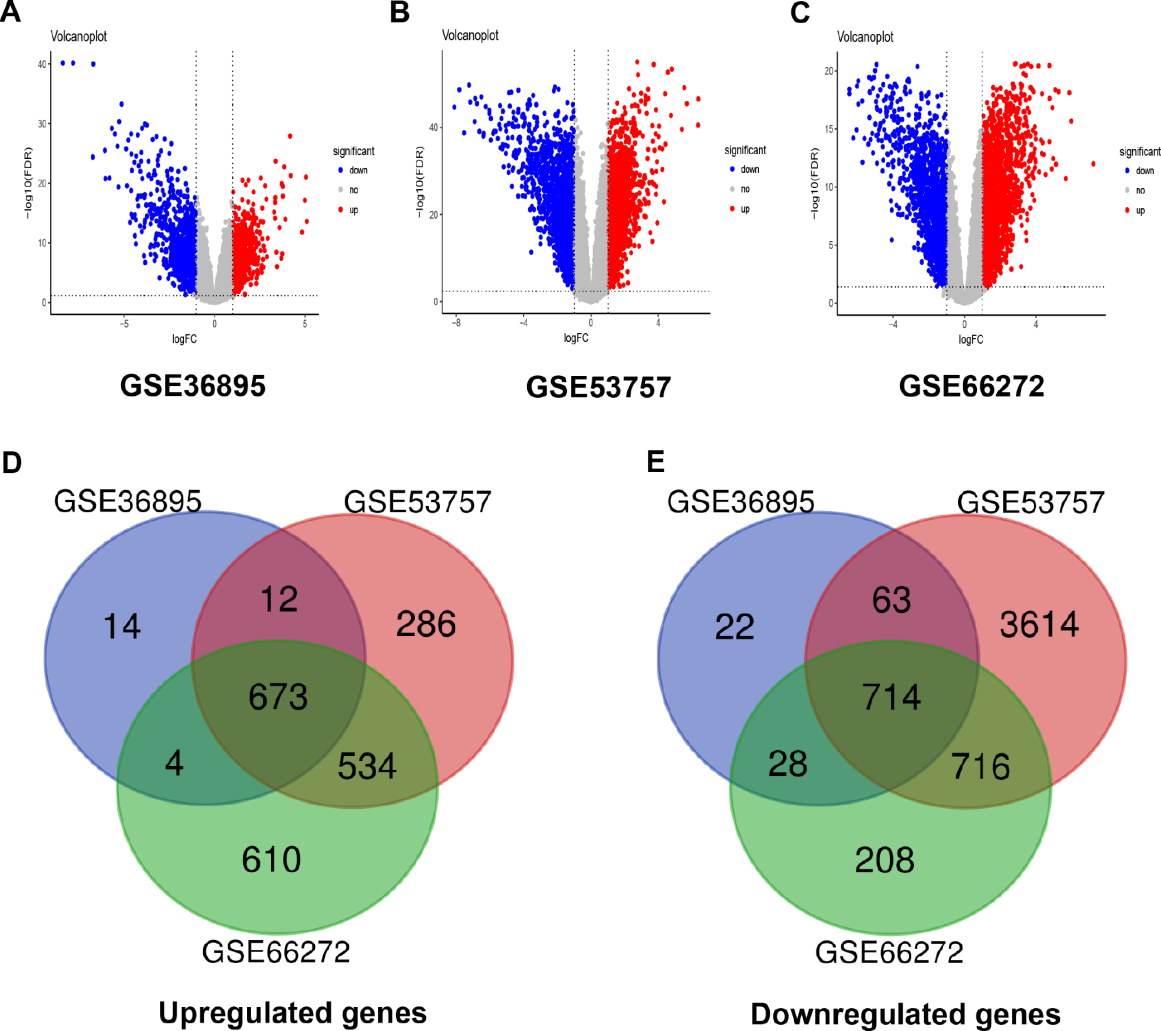
**Differentially expressed genes and common differentially expressed genes in three datasets.** (**A**–**C**) The volcano plots visualize the differentially expressed genes in GSE36895, GSE53757 and GSE66272, respectively. The red nodes represent upregulated genes. The green nodes represent downregulated genes. (**D**–**E**) Common differentially expressed genes in three datasets.

After data preprocessing and quality assessment, expression matrices of three expression profiles were obtained, including GSE36895, GSE53757 and GSE66272. Using |log2FC| > 1 and FDR < 0.05 as the threshold, all the differentially expressed genes were screened out in three expression profiles (Supplementary Tables 2–4). The DEGs of three datasets were shown as volcano plots in Figure 1A–1C. After being overlapped, the common 1387 genes were identified, including 673 upregulated and 714 downregulated genes (Figure 1D–1E).

### Weighted co-expression network construction and key modules identification

**Figure 2:**
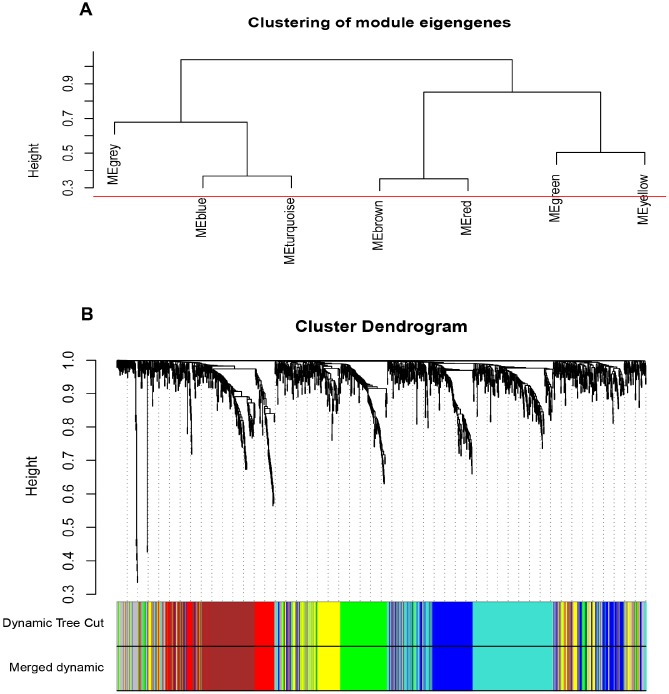
**Construction of WGCNA co-expression modules.** (**A**) The cluster dendrogram of module eigengenes. (**B**) The cluster dendrogram of the common differentially expressed genes in GSE66272. Each branch in the figure represents one gene, and every color below represents one co-expression module.

The input dataset for WGCNA construction consist of the common 1387 genes and 26 ccRCC samples with pathological stage and grade in GSE66272 (Supplementary Figure 2A). “WGCNA” package was used in R, after quality assessment for expression matrix of GSE66272, power of β = 8 (scale free R^2^ = 0.9) was selected to ensure a scale-free network (Supplementary Figure 2B–2E). Then we set MEDissThres as 0.25 to merge similar modules, and a total of 7 modules were identified (Figure 2A–2B). Blue module contained 247 genes, brown module contained 234 genes, green module contained 177 genes, red module contained 93 genes, turquoise module contained 301 genes, yellow module contained 187 genes, and 148 genes could not be included in any modules were put into the gray module, which was reserved for genes identified as not co-expressed. Genes in grey module were removed in the subsequent analysis.

### Interaction relationship of modules and identification of key modules

**Figure 3:**
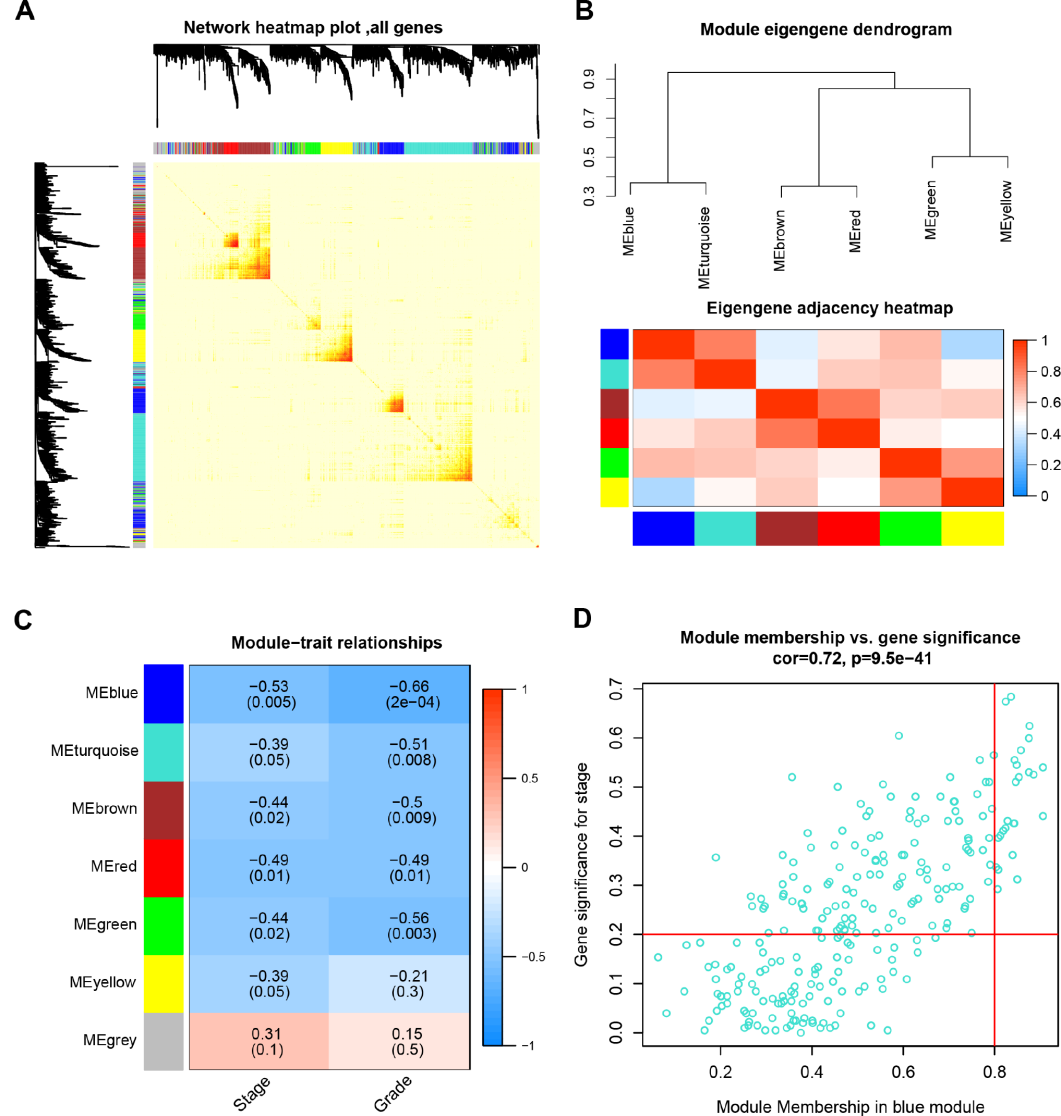
**Identification of modules associated with the clinical traits.** (**A**) Interaction relationship analysis of co-expression genes. Different colors of horizontal axis and vertical axis represent different modules. The brightness of yellow in the middle represents the degree of connectivity of different modules. There was no significant difference in interactions among different modules, indicating a high-scale independence degree among these modules. (**B**) Module eigengene dendrogram and eigengene network heatmap summarize the modules yielded in the clustering analysis. (**C**) Heatmap of the correlation between module eigengenes and pathological stage and grade. The blue module was significantly correlated with stage and grade. (**D**) Scatter plot of module eigengenes in blue module.

The network heatmap was performed to analyze the interaction relationship of 7 modules (Figure 3A). The results showed that each module was independent of each other, which indicated a high-scale independence degree among these modules and distinct independence of genes expression in each module. Then we calculated eigengenes of all modules and clustered them based on their correlation. Module eigengene dendrogram showed that the 6 modules were mainly divided into two clusters, and eigengene network heatmap demonstrated similar results (Figure 3B). Furthermore, the ME of the blue module showed the blue module was significantly associated with ccRCC tumor stage and grade compared with other modules (Figure 3C). Therefore, we selected the blue module for subsequent analysis, and identify the relevance between blue module and the clinical features with great biological significance (Supplementary Table 5).

### Functional annotation and KEGG pathway enrichment of blue modules

**Figure 4:**
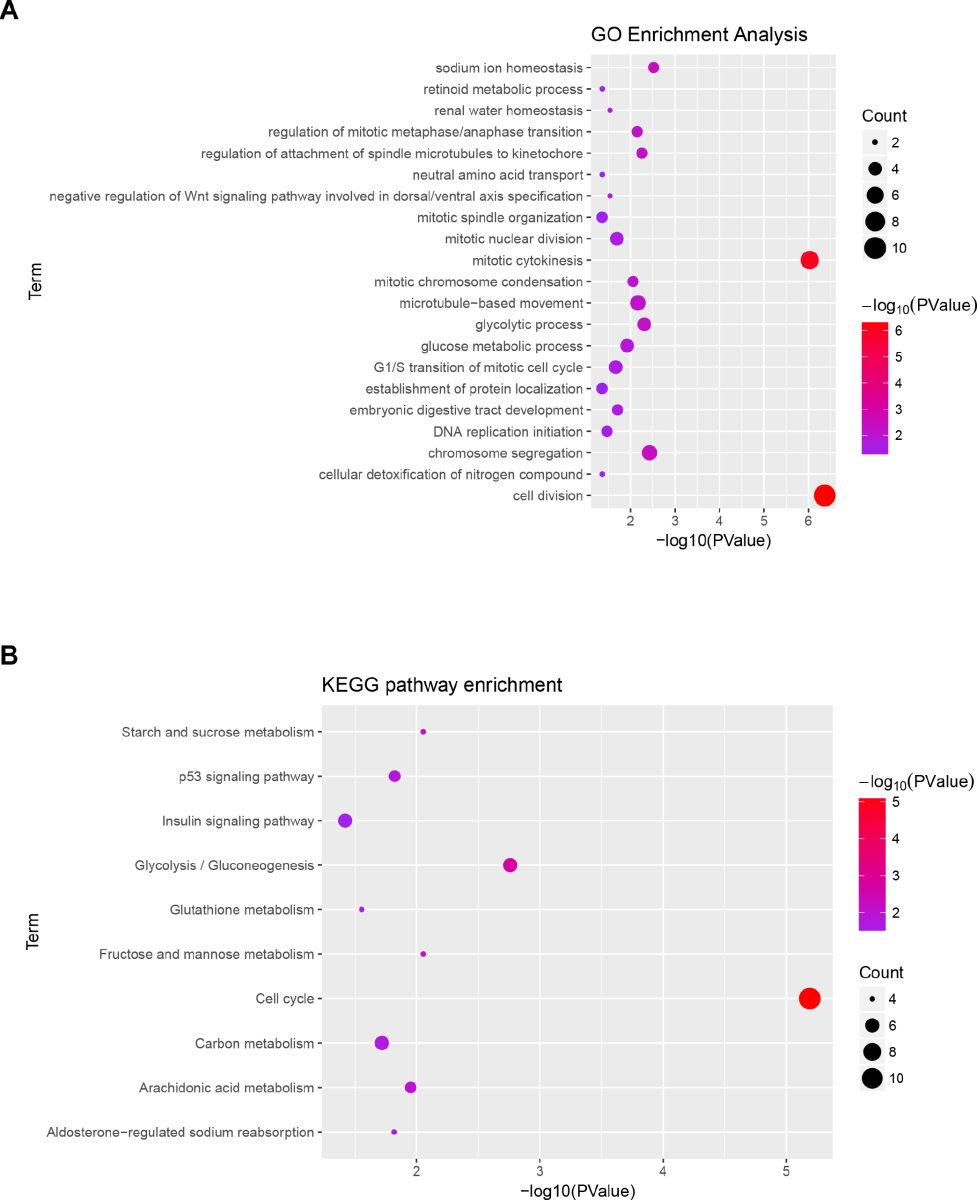
**Functional enrichment analysis of blue module.** (**A**) GO analysis of all genes in blue module. (**B**) KEGG pathway analysis of all genes in blue module.

Gene ontology analysis and KEGG pathway enrichment were performed for the above blue module to explore potential biological processes associated with ccRCC. Biological process of gene ontology analysis showed genes in the blue module were mainly associated with cell division, cell proliferation, cell cycle and metabolic related pathway (Figure 4A). The result of KEGG pathway enrichment was showed in Figure 4B. The most significant pathway was cell cycle, the other significant pathways included glycolysis/gluconeogenesis, fructose and mannose metabolism, starch and sucrose metabolism, arachidonic acid metabolism, p53 signaling pathway, aldosterone-regulated sodium reabsorption, carbon metabolism, glutathione metabolism and insulin signaling pathway.

### Hub genes detection and validation

**Figure 5:**
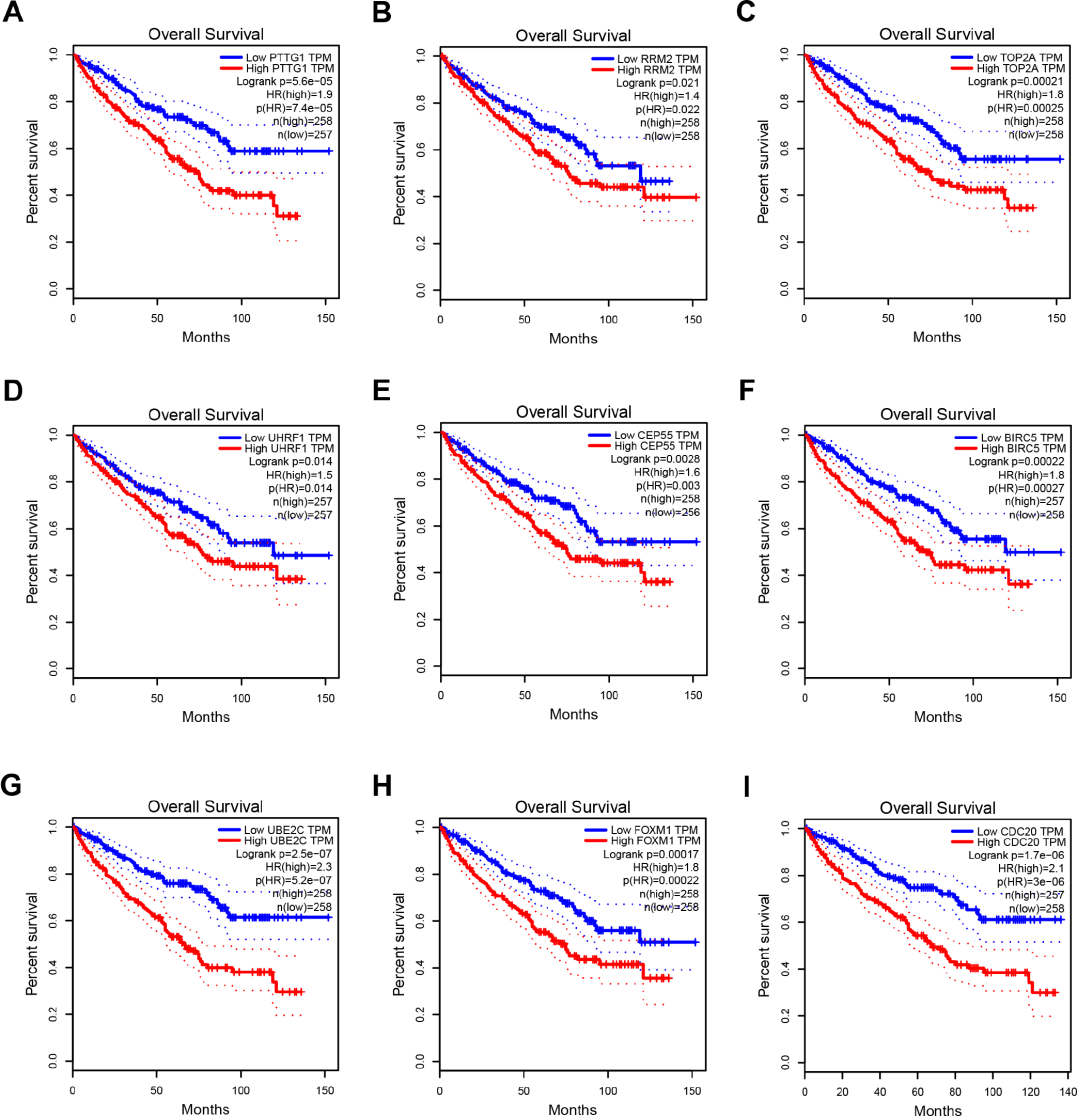
**Overall survival analysis of 9 key genes in ccRCC (based on TCGA data in GEPIA).** (**A**–**I**) Expression levels of PTTG1, RRM2, TOP2A, UHRF1, CEP55, BIRC5, UBE2C, FOXM1 and CDC20 are significantly related to the overall survival of patients with ccRCC (*P* < 0.05).

**Figure 6:**
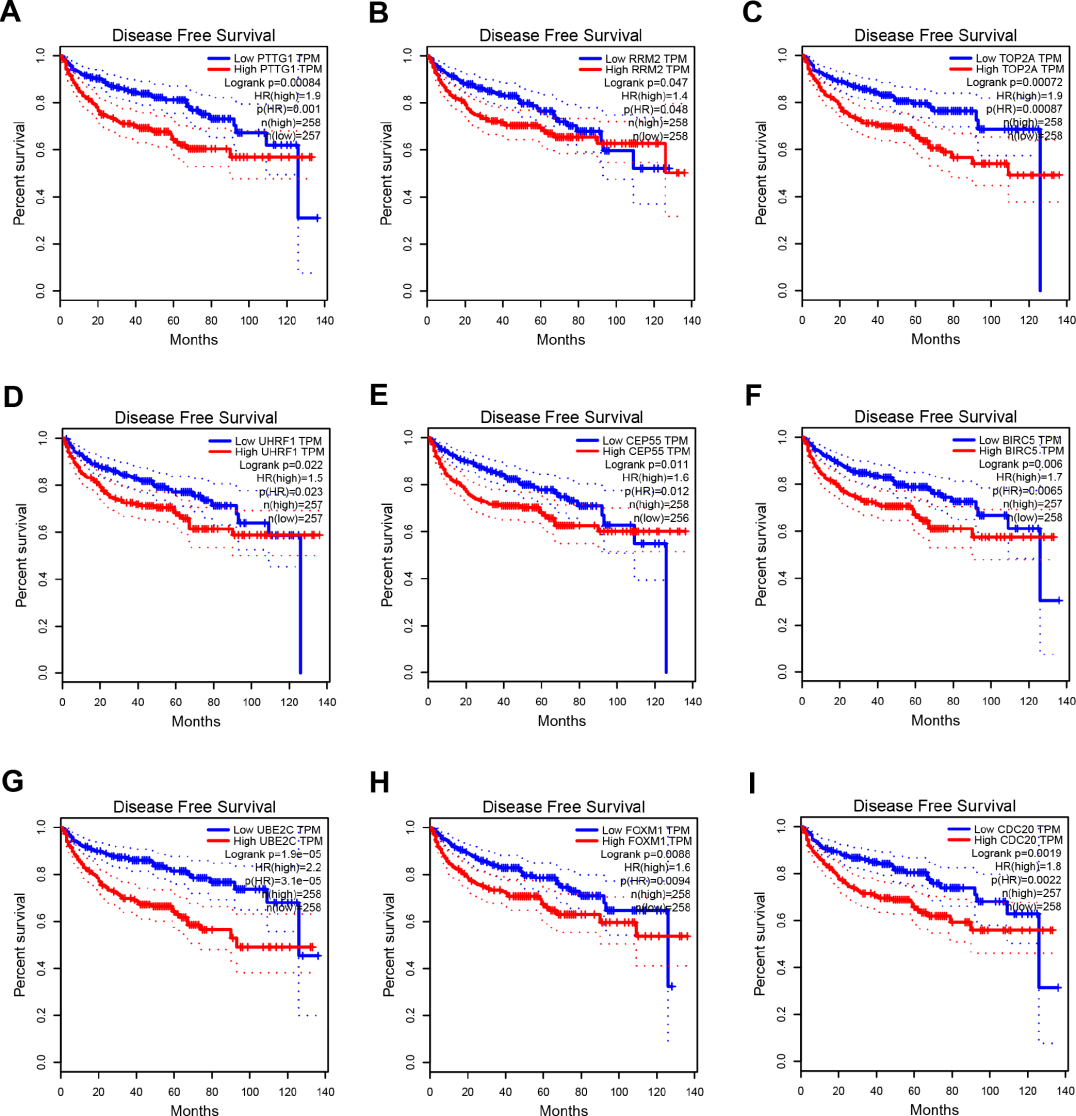
**Disease free survival analysis of 9 key genes in ccRCC (based on TCGA data in GEPIA).** (**A**–**I**) Expression levels of PTTG1, RRM2, TOP2A, UHRF1, CEP55, BIRC5, UBE2C, FOXM1 and CDC20 are significantly related to the disease free survival of patients with ccRCC (*P* < 0.05).

**Figure 7:**
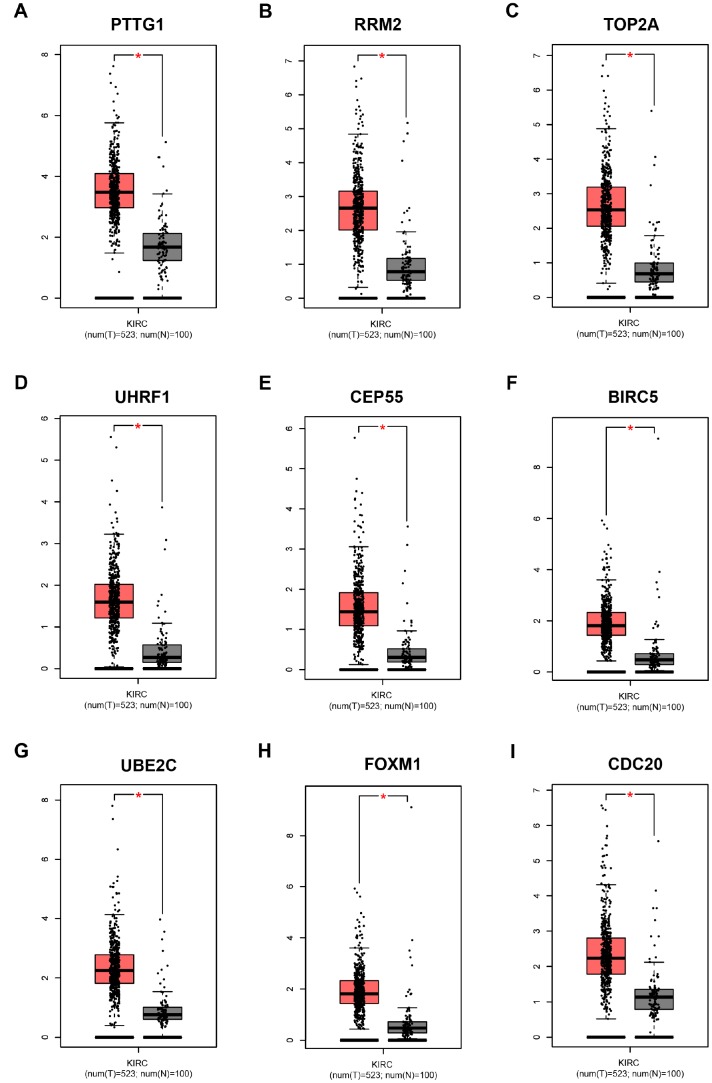
**Validation of the gene expression levels of PTTG1, RRM2, TOP2A, UHRF1, CEP55, BIRC5, UBE2C, FOXM1 and CDC20 between normal kidney and ccRCC tissues in GEPIA database.** (**A**–**I**) PTTG1, RRM2, TOP2A, UHRF1, CEP55, BIRC5, UBE2C, FOXM1 and CDC20 are significantly upregulated in ccRCC compared with normal tissues (*P* < 0.01). The red * represents *P* < 0.01.

**Figure 8:**
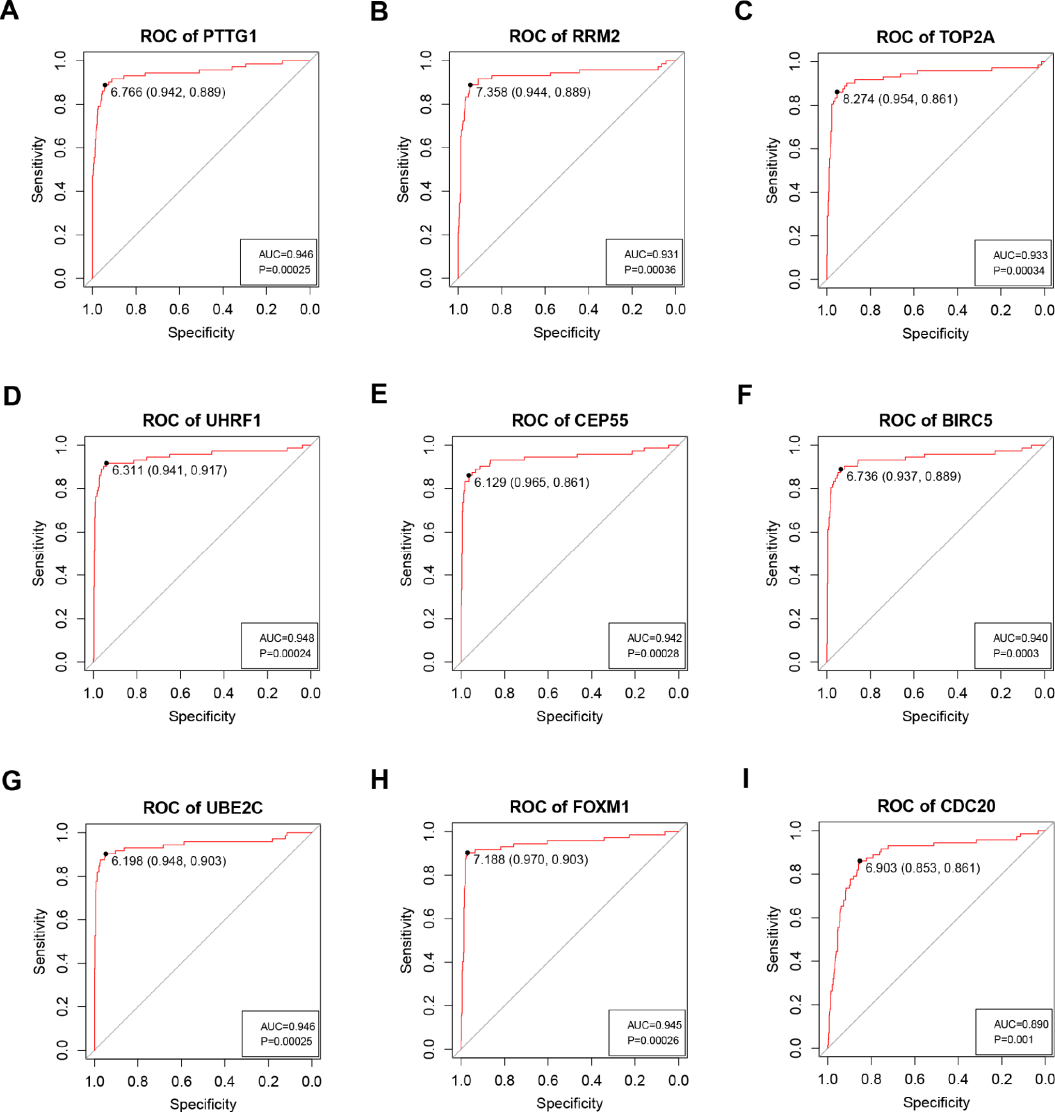
**ROC curve analysis of 9 key genes diagnosis. Receiver operating characteristic (ROC) curves and area under the curve (AUC) statistics are used to evaluate the capacity to discriminate ccRCC from normal controls with excellent specificity and sensitivity in TCGA dataset.** (**A**) PTTG1, (**B**) RRM2, (**C**) TOP2A, (**D**) UHRF1, (**E**) CEP55, (**F**) BIRC5, (**G**) UBE2C, (**H**) FOXM1, (**I**) CDC20.

Based on the criteria that cor.geneModuleMembership > 0.8 and cor.geneTraitSignificance > 0.2, 25 genes with the high connectivity in blue module were screened as hub genes (Figure 3D). Then 25 hub genes were validated using ccRCC data of GEPIA database. Among them, PTTG1, RRM2, TOP2A, UHRF1, CEP55, BIRC5, UBE2C, FOXM1 and CDC20 were negatively associated with the overall survival and disease free survival of ccRCC patients (Figures 5, 6). Moreover, based on the GEPIA database and Oncomine database, the expression levels of these 9 genes were significantly higher in ccRCC tumor tissues, compared with paracancerous normal tissues (Figure 7 and Supplementary Figure 3). In addition, based on individual cancer stage analysis, the expression of these 9 genes were significantly upregulated in the advanced tumor stages (Supplementary Figure 4). The protein expression levels of these 9 genes were significantly higher in tumor tissues compared with paracancerous normal tissues based on the Human Protein Atlas database (Supplementary Figure 5). A ROC curve was generated to verify the diagnostic performance of these 9 genes based on the TCGA database. The AUC showed that PTTG1, RRM2, TOP2A, UHRF1, CEP55, BIRC5, UBE2C, FOXM1 and CDC20 indicated excellent diagnostic efficiency for tumor and normal tissues (Figure 8). To further assess whether it can provide the favorable prognostic value based on these 9 gene expression levels, the multivariate Cox regression analysis was performed. The results in Table 1 showed that the risk score base on these 9 genes signature was a clinically independent prognostic factor for ccRCC patients.

**Table 1:** Multivariate Cox regression analysis of potential prognostic factors for ccRCC patients.

**Variables**	**Overall survival**			**Disease free survival**		
	**HR**	**95%CI of HR**	***P***	**HR**	**95%CI of HR**	***P***
**Risk score**	1.26	1.10-1.43	0.00054	1.18	1.01-1.39	0.038
**Age**	1.50	1.10-2.05	0.01	1.14	0.78-1.66	0.50
**Gender**	1.18	0.85-1.63	0.32	0.80	0.54-1.21	0.29
**Grade**	1.37	1.08-1.73	0.0094	1.67	1.27-2.20	0.0002
**Stage**	1.60	1.37-1.86	1.76e-09	2.29	1.90-2.77	2e-16

### Gene set enrichment analysis (GSEA)

GSEA was performed to explore biological function of 9 hub genes. We seted the cut-off criteria as gene size ≥ 10, FDR < 0.05, and |enrichment score (ES)| > 0.65, the results revealed that high expression samples in all 9 key genes were enriched in cell cycle pathway (Supplementary Figure 6).

### Genetical alteration of hub genes

**Figure 9:**
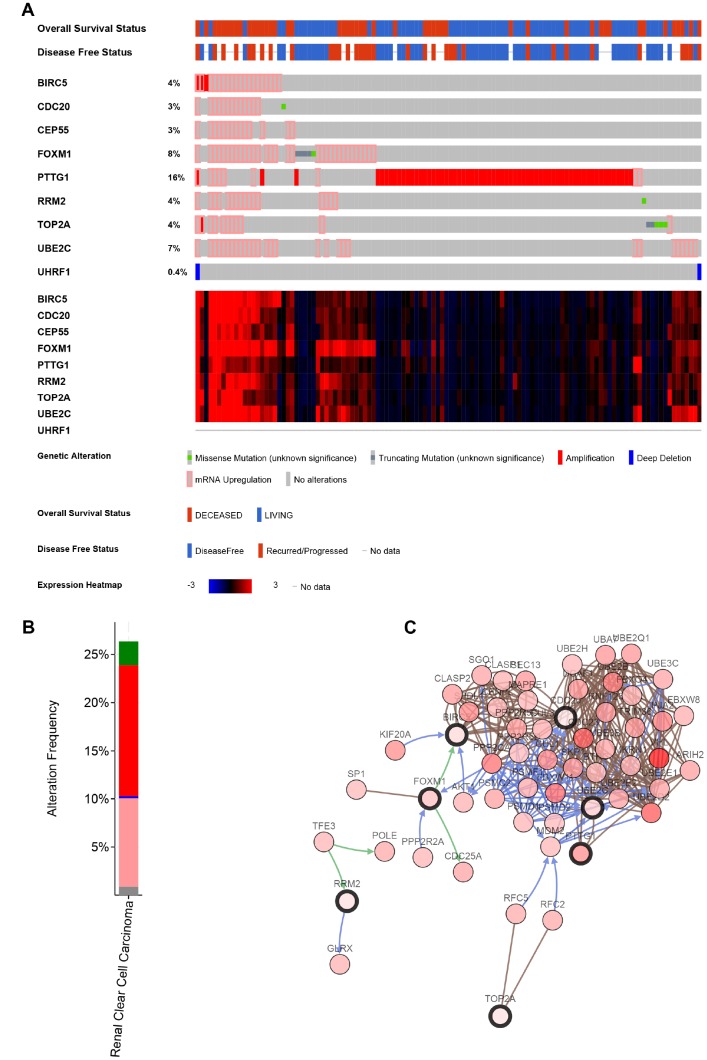
**Genetic alterations associated with 9 key genes.** (**A**) A visual summary of Genetic alterations (data from ccRCC in TCGA, provisional) shows the genetic alteration of 9 key genes which were altered in 118 (26%) of 446 ccRCC patients. (**B**) The total alteration frequency of 9 key genes is illustrated. (**C**) The network contains 59 nodes, including 9 key genes and the 50 most frequently altered neighbor genes. Relationship of 9 key genes is also illustrated.

The alteration statuses of 9 key genes were analyzed using TCGA ccRCC patients’ data of cBioPortal database. The 9 hub genes altered in 118 (26%) of 446 ccRCC patients (Figure 9B), and the frequency of alteration of each hub gene was shown in Figure 9A. PTTG1 and FOXM1 altered most (16% and 8%, respectively), amplification and mRNA upregulation were the main type. Figure 9C showed the relationship of the 9 genes and the other 50 most frequently altered neighbor genes. FOXM1, BIRC5, CDC20 and UBE2C were significantly important in the network.

### Related small molecule drugs screening

To identify candidate small molecule of ccRCC, CMap database was performed to screen out small molecule drugs. According to analyze consistent differently expressed probesets between ccRCC samples and adjacent normal samples, the related small molecule drugs with highly significant correlations were identified. 10 small molecule drugs were filter by the number of instances (n > 10) and *P* value (< 0.05). They were listed in Table 2. Among these small molecule drugs, trichostatin A (TSA), trifluoperazine, genistein and prochlorperazine showed higher negative correlation and the potential to treat ccRCC.

**Table 2:** Results of CMap analysis.

**Rank**	**cmap name**	**mean**	**n**	**enrichment**	***P***	**specificity**	**percent non-null**
1	trichostatin A	-0.375	182	-0.227	0	0.7525	75
2	trifluoperazine	-0.466	16	-0.46	0.00128	0.1635	75
3	genistein	0.332	17	0.436	0.00208	0.0523	64
4	prochlorperazine	-0.492	16	-0.44	0.00243	0.0849	87
5	tanespimycin	-0.291	62	-0.222	0.00385	0.5714	59
6	vorinostat	-0.497	12	-0.462	0.00692	0.4602	91
7	chlorpromazine	-0.397	19	-0.369	0.00774	0.0321	73
8	alpha-estradiol	-0.483	16	-0.385	0.01219	0.224	87
9	LY-294002	-0.292	61	-0.201	0.01252	0.638	68
10	clozapine	0.086	17	0.322	0.04636	0.1156	52

## DISCUSSION

ccRCC is a heterogeneous tumor. The occurrence and progression of ccRCC are the comprehensive results activation of various oncogenes and inactivation of various tumor suppressor genes. In the present study, we used comprehensive bioinformatics analysis to identify 9 key genes associated with progression and prognosis of ccRCC patients, and select several new small molecule drugs that have the potential to treat ccRCC.

The 9 key genes consist of BIRC5, CDC20, CEP55, FOXM1, PTTG1, RRM2, TOP2A, UBE2C and UHRF1. They were all oncogenes, and associated with progression and prognosis of ccRCC patients. Few of them were identified as biomarkers in clear cell renal cell carcinoma. BIRC5 (survivin) is a member of the IAPs family. It can suppress apoptosis and regulate cell proliferation. BIRC5 overexpression has been reported in various malignancies, and it was a prognostic marker in renal cell carcinoma [5–7]. Two meta-analysis suggested that high survivin expression was associated with poor prognosis and more advanced pathological stage, and it could be used as a biomarker for disease management [8, 9]. CDC20 is one of the cell cycle related proteins. It was high expressed in most malignant tumor tissues and played an oncogenic role in tumorigenesis and tumor progression. Wu *et al.* reported that CDC20 expression was an independent prognostic factor in colorectal cancer and can serve as a potential prognostic biomarker [10]. Gao *et al.* also found that the growth and invasion of osteosarcoma cells was restrained by inhibiting CDC20 expression [11]. CEP55 is a member of the coiled-coil protein family, its main function is to anchor microtubule-associated proteins, participate in spindle formation, and regulate cell proliferation [12]. CEP55 is expressed in normal tissues and tumor cells, and is coupled with centrosomes and intermediates in the cell cycle, and plays a role in regulating cell cycle after phosphorylation. It has been found that CEP55 overexpression is significantly associated with tumor stage, invasiveness, and tumor metastasis of many malignant tumors [13–15]. FOXM1 is a transcription factor. It plays an important role in the regulation of multiple biological processes, including cell proliferation, cell cycle progression, cell differentiation, DNA damage Repair, tissue homeostasis, angiogenesis and apoptosis. Some research results indicated that FOXM1 plays a major role in tumorigenesis, Tan *et al.* found that FOXM1 was a specific marker in triple-negative breast cancer [16]. Breyer *et al.* identified that FOXM1 expression was associated with advanced clinical and pathological feature in bladder cancer [17]. PTTG1 is an oncogene which is closely associated to cell proliferation, differentiation and various signal transduction pathways. PTTG1 can directly induce carcinogenesis by cell transformation, activating proto-oncogenes and growth factors [18]. RRM2 is a key enzyme in DNA synthesis and repair pathways, and high expression of RRM2 is relative to tumor angiogenesis, invasion and metastasis [19, 20]. Previous literature reported that RRM2 promoted tumorigenesis and progression of pancreatic cancer, lung cancer, gastric cancer, ovarian cancer, bladder cancer and other tumors [21–24], TOP2A gene encoded a DNA topoisomerase, it is an ATP-dependent synthetase and hydrolase that plays a key role in cells and plays an important role in many cellular biological processes, such as DNA replication, chromatin condensation, chromosome segregation, and chromosome structure retention. Studies have found that high expression of TOP2A promoted the progression of breast cancer [25, 26]. UBE2C is also known as UbcH10, which is a member of the ubiquitin-coupled enzyme E2 family. It has been reported that the expression level of UBE2C is positively correlated with tumor grade and poor prognosis in the adrenal cancer, breast cancer, colon cancer, lung cancer and ovarian cancer [27–31]. UHRF1 is a newly discovered oncogene which related to cell growth. As a an important epigenetic regulator, it plays an important role in the maintenance of DNA methylation, and participates in important biological processes such as cell proliferation, cell cycle regulation, apoptosis and radiosensitivity, regulating cell cycle G1-S phase and G2-M phase transition, thereby promoting tumor progression [32].

The multivariate Cox regression results showed that these 9 key genes selected in our study may also represent candidate biomarkers for predicting prognosis of ccRCC patients. To further explore potential mechanism of 9 hub genes, we performed GSEA analysis of all 9 hub genes. The results revealed that all hub genes were significantly enriched in terms of cell cycle pathway. Several researchers had reported that Cell cycle disorder is the most important mechanism of tumors. In the regulation of the cell cycle, abnormalities of various molecules may cause tumorigenesis and progression. Thus, we might suppose that 9 hub genes played key role in the tumorigenesis and progression of ccRCC probably by regulating cell cycle pathway, which contributed to the poor prognosis of ccRCC.

In addition, we used CMap database to identify several small molecule drugs with potential therapeutic efficacy against ccRCC. Some of them in our results have been proven to have anti-cancer effects, such as TSA and trifluoperazine. TSA is a histone deacetylase (HDAC) inhibitor, which shows a potential therapeutic effect in various types of cancer cells, when combined with radiotherapy or chemotherapy. Trifluoperazine is a typical antipsychotic, but recently some researchers found that trifluoperazine could inhibit the proliferation of multiple cancer cells, such as glioblastoma, Hepatocellular Carcinoma and lung cancer [33–35]. Thus, we might consider that these identified molecule drugs could have potential to treat ccRCC. So our research may provide some potential biomarkers or molecular targets for ccRCC.

However, this study has some limitations. Firstly, this is a retrospective study, all the data of this study were obtained from publicly available database. a multicenter and prospective study is needed to evaluate the possible applications of molecular signatures to predict survival. Secondly, further studies including in vivo and in vitro experiments are needed to elucidate molecular mechanisms of key genes for clinical applications.

In conclusion, using weighted gene co-expression analysis, our study identified 9 key genes associated with progression and prognosis, which can provide the favorable prognostic value based on these 9 gene expression levels, and several candidate small molecule drugs that had the potential to treat ccRCC tumors in ccRCC tumors, which provide direction for ccRCC tumors targeted therapy.

## MATERIALS AND METHODS

### Gene expression profiles data

Three gene expression profiles of mRNA and related clinical data of ccRCC were downloaded from Gene Expression Omnibus (GEO) database (http://www.ncbi.nlm.nih.gov/geo/) (Supplementary Table 1). GSE36895 includes 29 ccRCC tissues and 23 normal tissues, GSE53757 includes 72 ccRCC tissues and 72 normal tissues, and GSE66272 includes 26 ccRCC tissues and 26 adjacent normal tissues. Three gene expression profiles were used to screen differentially expressed genes. GSE66272 was performed to construct weighted gene co-expression networks analysis for this study. Gene sequencing data and corresponding clinical information of ccRCC were obtained from The Cancer Genome Atlas (TCGA) data portal (https://portal.gdc.cancer.gov/), which were used for validation of hub genes. A flowchart of this study was showed in Supplementary Figure 1.

### Data preprocessing and differentially expressed genes (DEGs) screening

For the microarray analyses, RMA method was used for background correction of raw gene expression matrixes, then log2 transformation of expression matrixes. the “affy” R package was utilized for quantile normalization, median polish algorithm summarization [36]. Then all gene probes were mapped into gene symbols by the affymetrix annotation files. The “limma” (linear models for microarray data) R package was performed for DEGs identifying between ccRCC samples and normal kidney samples. Cut-off criteria for screening DEGs were false discovery rate (FDR) < 0.05 and |log2fold change| ≥ 1. For TCGA ccRCC data, the gene expression data were based on the RNA-sequencing technology of IlluminaHiseq. The read counts were used to represent the genes expression level. Data processing was performed as we described before [37]

### Weighted co-expression network analysis

Weighted gene co-expression network were constructed by “WGCNA” R package, as previously described [38, 39]. First, sample clustering of common DEGs was performed to check if they were good genes and good samples. Second, a soft threshold power β was selected in accordance with standard scale-free networks. Third, we calculated the adjacencies between all filtered genes by the power adjacent function to Pearson correlation matrix to transform data into a topological overlap matrix (TOM), and the corresponding dissimilarity (1-TOM) was calculated. Then, According to the TOM-based dissimilarity measurement, average linkage hierarchical clustering was conducted with a minimum size of 50 for the genes dendrogram. To further analyze the module, the dissimilarity of module eigengenes was calculated. Highly similar modules were identified by clustering and then merged together with a height cut-off of 0.25.

### Identification of clinically significant modules and functional annotation

The module eigengene (ME) was defined as the first principal component of a given module. It could be regarded as a representative of the gene expression profiles from a module, the ME can summarize the gene expression profiles, the correlation between ME and clinically significant trait was calculated to identify the relevant module. Gene significance (GS) was defined as the log10 transformation of the *P* value (GS = lgP) in the linear regression between gene expression and pathological progression, and module significance (MS) represented the average GS for all the genes in a module. In general, the module with the absolute MS ranked first among all the selected modules was considered as the one related with clinical trait. In order to explore the potential mechanism of how module genes impact correlative clinical feature, we uploaded all genes in blue module into Database for Annotation, Visualization, and Integrated Discovery (DAVID) (http://david.abcc.ncifcrf.gov/) online tool [3]. GO functional enrichment analysis and Kyoto Encyclopedia of Genes and Genomes (KEGG) pathway enrichment were performed. *P* value < 0.05 was set as the cutoff criteria.

### Hub genes detection and validation

The genes with the maximum intramodular connectivity were defined as hub genes. Firstly, the most significant module was identified. Then, hub genes were screened according to the criteria that cor.geneModuleMembership > 0.8 and cor.geneTraitSignificance > 0.2. Further, the differential expression of hub genes in ccRCC was validated using Gene Expression Profiling Interactive Analysis (GEPIA) database, Oncomine and Human Protein Atlas database. ROC curve was performed to verify the diagnostic performance of hub genes. Kaplan-Meier survival curve of overall survival and disease free survival was used to analyze survival differences. In addition, the selected 9 hub genes were put in a multivariate Cox regression analysis. Risk score of 9 hub genes was developed based on the mRNA expression level weighted by the estimated regression coefficient in the multivariate Cox regression analysis. The risk score for each patient was calculated as follows, risk score $=\sum_{i=1}^{n}\left(coef_{i}\text{*}\;{\text{Expr}}_{i}\right)$, where was the expression of the genes in the signature for patient i, coef i is the Cox coefficient of the genes i.

### Genetical alteration of hub genes

The cBioPortal for Cancer Genomics (http://www.cbioportal.org/) is a large-scale cancer genomics database [40]. It provides an open platform to explore, visualize and analyze multi-dimensional cancer genomic data. Researchers can interactively explore the genetic changes of different samples, genes, and paths. This site also provides gene level graphical summaries from multi-platform, web visualization analysis and survival analysis. We used cBioPortal to explore genetic alterations connected with the 9 hub genes and their correlation with other famous genes.

### Gene set enrichment analysis (GSEA)

GSEA (http://software.broadinstitute.org/gsea/index.jsp) was used to explore biological function of 9 hub genes [41]. Annotated gene sets c2.cp.kegg. v5.2.symbols.gmt was chosen as the reference gene sets. Gene size ≥ 10, FDR < 0.05, and |enrichment score (ES)| > 0.65 were set as the cut-off criteria.

### Identification of candidate small molecules

Connectivity map (CMap) is a gene expression profiles database. It is constructed by team led by Todd Golub and Eric Lander [42]. Firstly, small molecule drugs were utilized to process human cells. Then, differential expressed genes after treatment were used to establish a database, which interrelated small molecule drugs, gene expression and disease. It could help researchers to quickly identify molecule drugs with high correlation with diseases, the chemical structure of molecule drugs and the possible mechanism of molecule drugs.

## Supplementary Material

Supplementary Figures

Supplementary Tables

Supplementary Table 2

Supplementary Table 3

Supplementary Table 4

Supplementary Table 5
