# Supplementary material for: Identification of 9 key genes and small molecule drugs in clear cell renal cell carcinoma
Source: Aging (Albany NY). 2019 Aug 18;11(16):6029–52. doi: 10.18632/aging.102161 (PMC6738436; doi:10.18632/aging.102161)
Supplement: Supplementary Figures [file aging-11-102161-s006.pdf]

SUPPLEMENTARY FIGURES

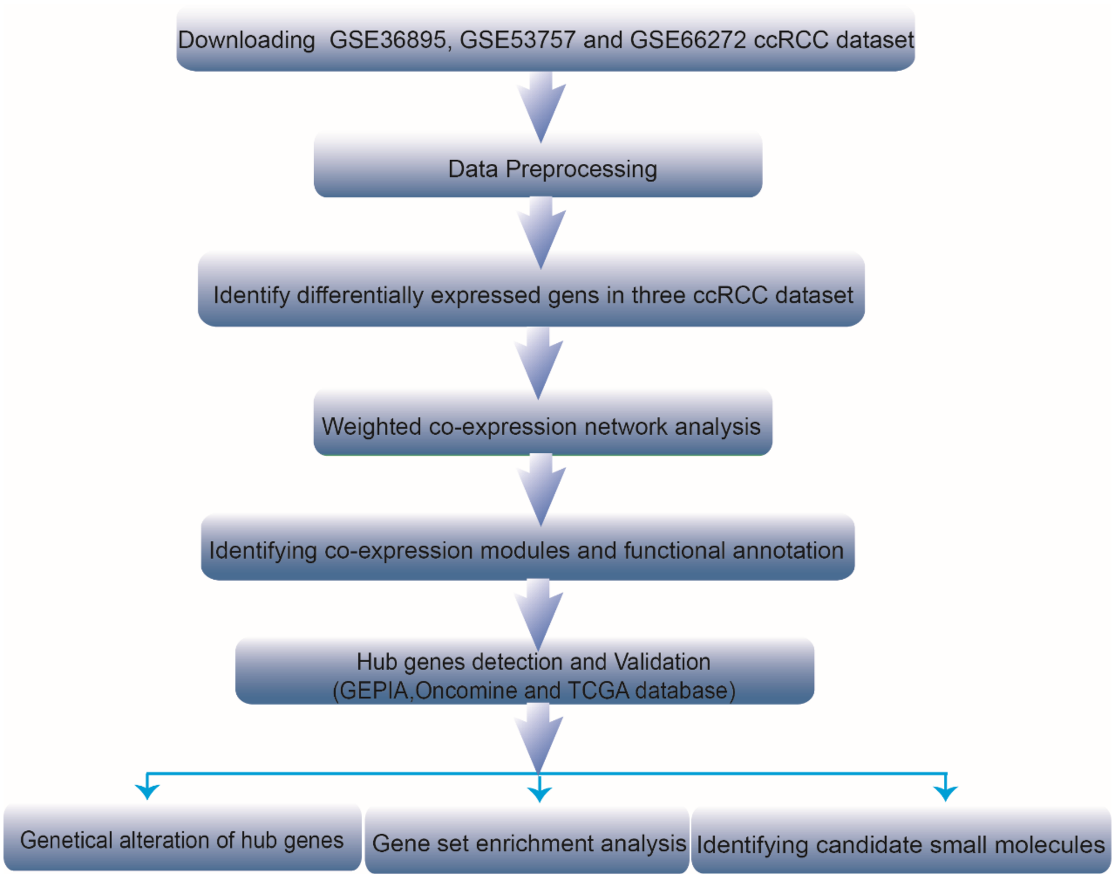

Supplementary Figure 1. Flow chart of data preparation, processing, analysis and validation in this study.

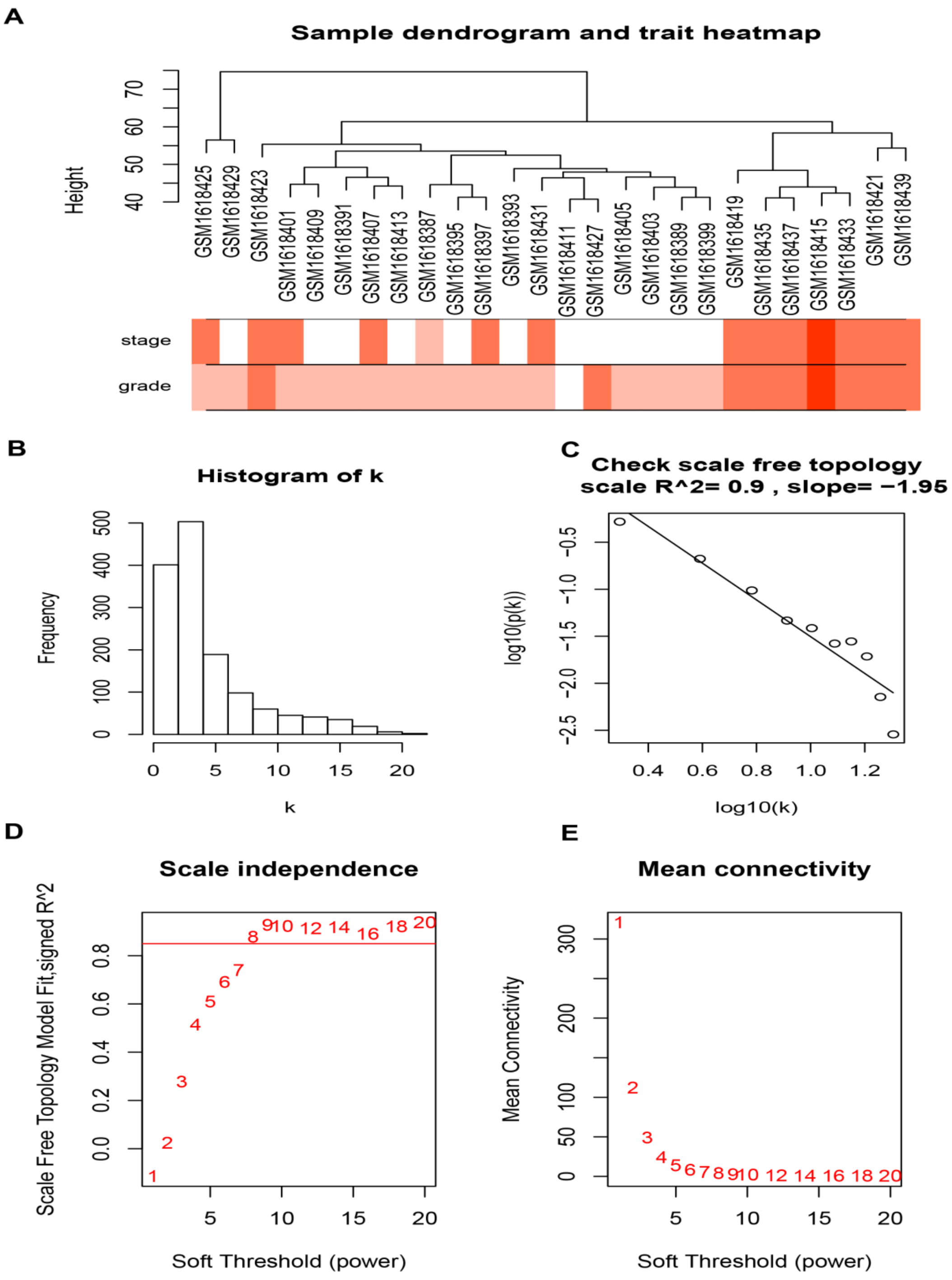

Supplementary Figure 2. Clustering dendrogram of ccRCC tumor samples and the clinical traits, and determination of soft-thresholding power. (**A**) The clustering was based on the common differentially expressed genes in ccRCC. The red color represented stage and grade. The color intensity was proportional to higher pathological stage and grade. (**B**) Analysis of the scale-free fit index for various soft-thresholding powers (β). (**C**) Analysis of the mean connectivity for various soft-thresholding powers. (**D**) Histogram of connectivity distribution when β = 8. (**E**) Checking the scale free topology when β = 8.

Analysis Type by Cancer	PTTG1		RRM2		TOP2A		UHRF1		CEP55		BIRC5		UBE2C		FOXM1		CDC20	
	Cancer vs. Normal		Cancer vs. Normal		Cancer vs. Normal		Cancer vs. Normal		Cancer vs. Normal		Cancer vs. Normal		Cancer vs. Normal		Cancer vs. Normal		Cancer vs. Normal	
Bladder Cancer	5		6	1	9		1		4		3		6		4		6	
Brain and CNS Cancer	7	1	12	1	15	1	10	1	7	1	12	1	14	1	14	1	8	2
Breast Cancer	30	1	19	1	31	1	21	1	24	1	16	1	31		17		21	1
Cervical Cancer	3		4		5		2		4		3		3		4		3	
Colorectal Cancer	11		18		23		15		20		12		20		9		9	
Esophageal Cancer	2	2	3		5		2		6		2		6		5		3	
Gastric Cancer	6		5		11		5		7		5	1	9		7		5	
Head and Neck Cancer	6		9		12		5		11		6		6		7		9	
Kidney Cancer	3		4		5		1		5	1	2	5	3		2		1	
Leukemia	1	3	2	11	3	10	8	4	2	2	4	8		6	1	3	1	9
Liver Cancer	4		5		4		1		2		3	1	4		4		3	
Lung Cancer	15		15		21	1	5		11		12	2	19	1	15		12	1
Lymphoma	12	1	22	1	12	2	6		9		16		9		13		12	2
Melanoma	3		2		3	1	1		3		3		2		2		2	
Myeloma	1		2		2				1		1							2
Other Cancer	5	10	9		11	5	6	1	7	7	7	2	13		10	1	6	2
Ovarian Cancer	4		5		7		3		5		3		8		6		4	
Pancreatic Cancer	5		2		6	1	2		3		2	1	2		3		3	
Prostate Cancer			2		3		2		1		4		1					
Sarcoma	11		11		14				9		13		11	1	12		12	1
Significant Unique Analyses	132	18	155	15	200	22	96	7	139	12	126	22	166	9	134	5	118	20
Total Unique Analyses	422		449		462		291		387		464		450		455		415	

Supplementary Figure 3. Gene expression level of 9 key genes (based on Oncomine database).

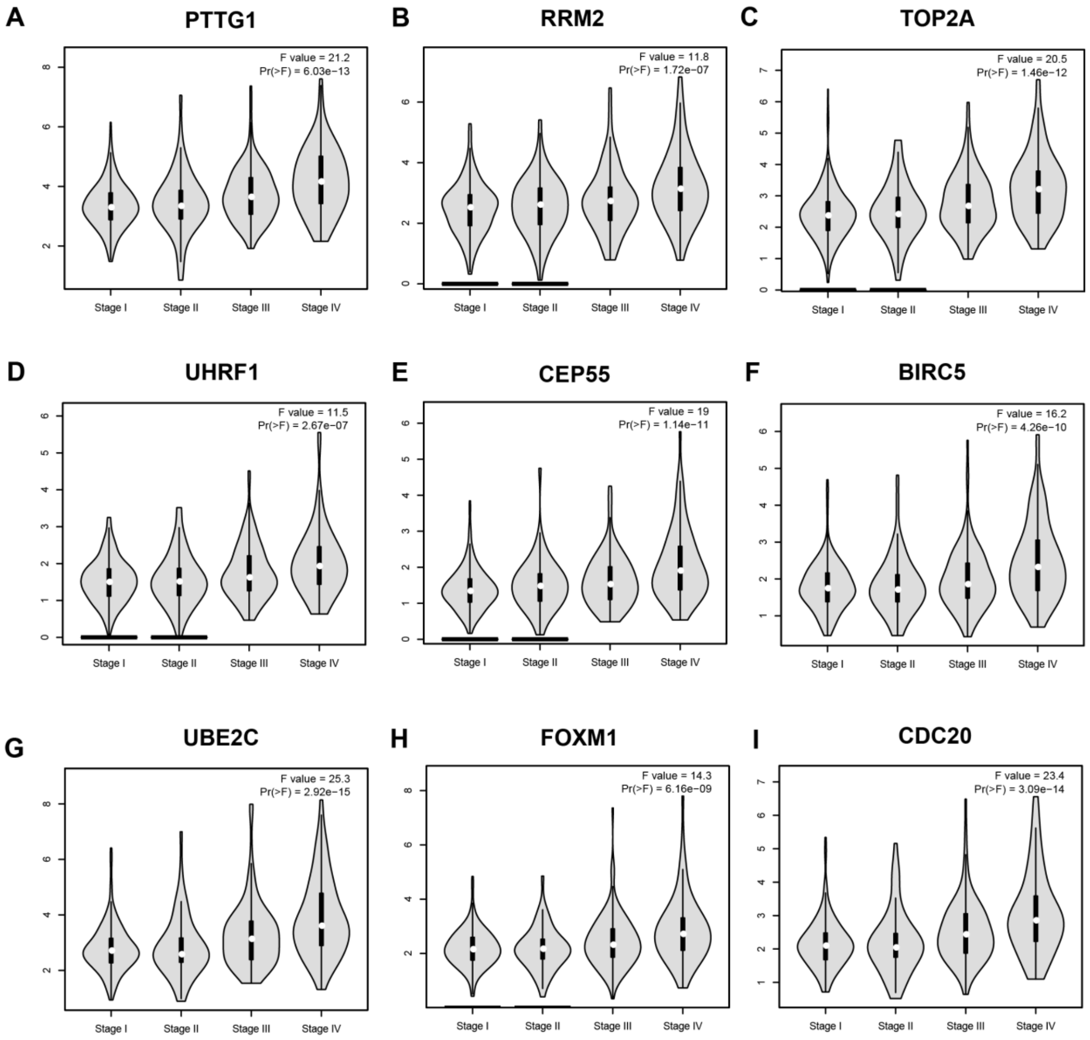

Supplementary Figure 4. Validation of the correlation between 9 key genes expression levels and the pathologic stages of ccRCC (based on ccRCC data in GEPIA database). (**A**) PTTG1, (**B**) RRM2, (**C**) TOP2A, (**D**) UHRF1, (**E**) CEP55, (**F**) BIRC5, (**G**) UBE2C, (**H**) FOXM1 and (**I**) CDC20.

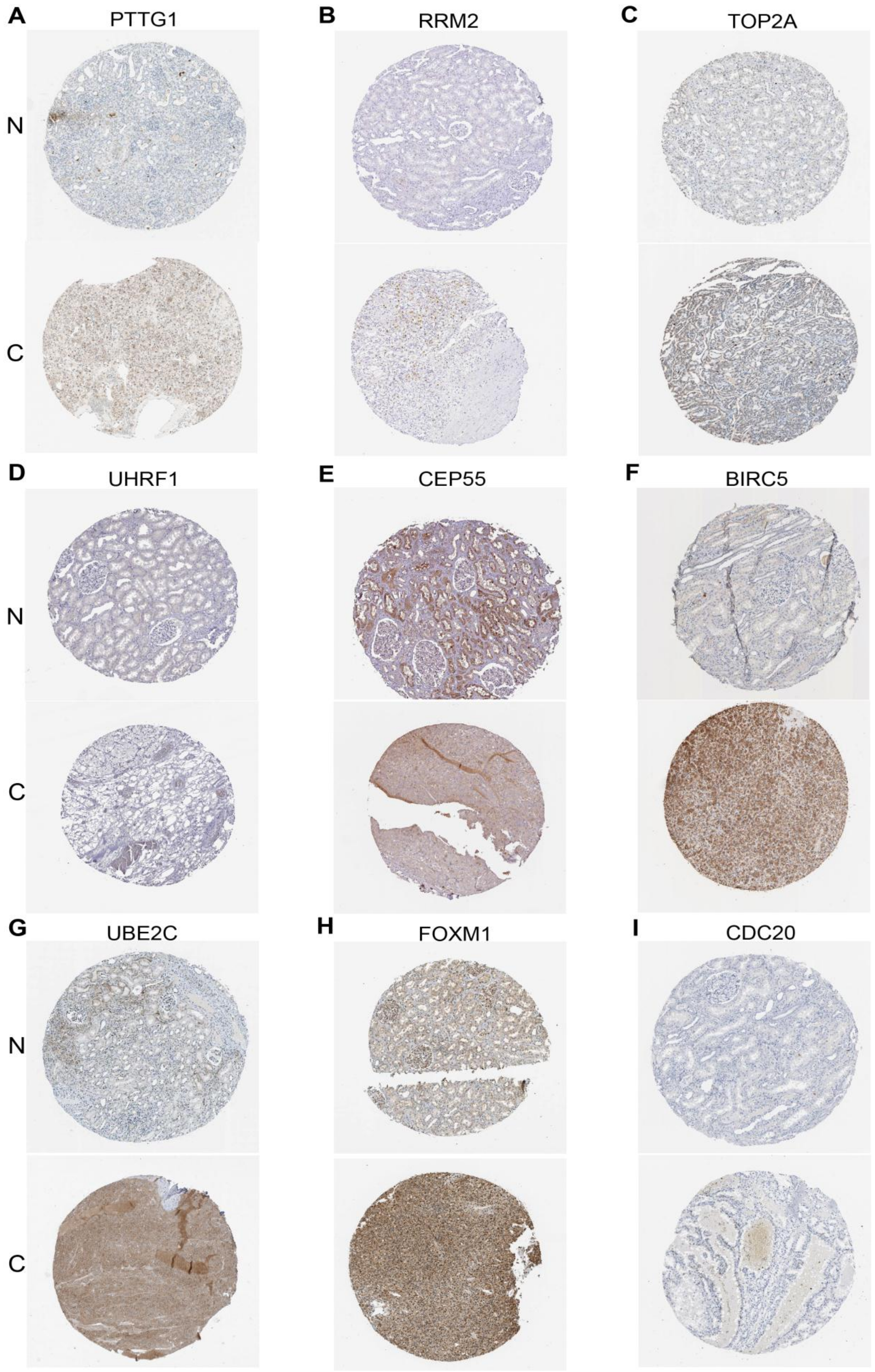

Supplementary Figure 5. Immunohistochemistry of the 9 key genes based on the Human Protein Atlas. (**A**) PTTG1, (**B**) RRM2, (**C**) TOP2A, (**D**) UHRF1, (**E**) CEP55, (**F**) BIRC5, (**G**) UBE2C, (**H**) FOXM1, (**I**) CDC20.

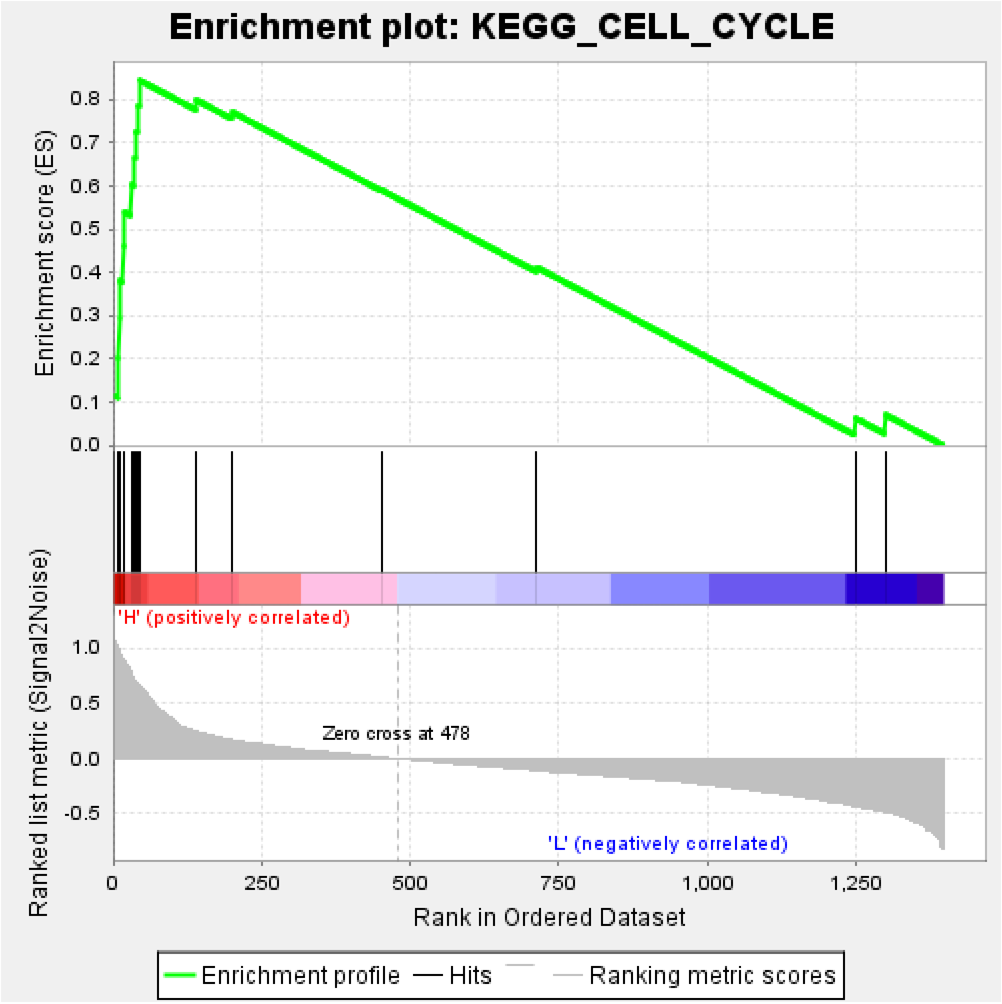

Supplementary Figure 6. Gene set enrichment analysis (GSEA). Only listed the common functional gene sets enriched in ccTCC with 9 key genes highly expressed.
