# Supplementary material for: Identification of 9 key genes and small molecule drugs in clear cell renal cell carcinoma
Source: Aging (Albany NY). 2019 Aug 18;11(16):6029–52. doi: 10.18632/aging.102161 (PMC6738436; doi:10.18632/aging.102161)
Supplement: Supplementary Tables [file aging-11-102161-s003.pdf]

SUPPLEMENTARY TABLES

Supplementary Table 1. The detailed information of the expression profile datasets.

Dataset	Array types	Experiment type
GSE36895	[HG-U133_Plus_2] Affymetrix Human Genome U133 Plus 2.0 Array	Expression profiling by array
GSE53757	HG-U133_Plus_2] Affymetrix Human Genome U133 Plus 2.0 Array	Expression profiling by array
GSE66272	HG-U133_Plus_2] Affymetrix Human Genome U133 Plus 2.0 Array	Expression profiling by array
The Cancer Genome Atlas (TCGA)	Illumina HiSeq platform	RNA-sequencing profiling

Please browse Full Text version to see the data of Supplementary Tables 2 and 5.

Supplementary Table 2.

Supplementary Table 3.

Supplementary Table 4.

Supplementary Table 5.
